# Incidence and risk factors of clinically important venous thromboembolism in tibial plateau fractures

**DOI:** 10.1038/s41598-022-24717-1

**Published:** 2022-11-23

**Authors:** Pengfei Wang, Xinan Yan, Chen Fei, Binfei Zhang, Jian Xing, Kun Zhang, Utku Kandemir

**Affiliations:** 1 Department of Orthopedics and Traumatology, Honghui Hospital, Xi’an Jiaotong University, Xi’an, China; 2 Department of Orthopaedic Surgery, University of California, San Francisco, CA USA; 3 Department of Orthopedics and Traumatology, Honghui Hospital, Xi’an Jiaotong University Health Science Center, Xi’an, China

**Keywords:** Trauma, Thrombosis

## Abstract

While there are multiple reports on venous thromboembolism (VTE) associated with several orthopedic procedures, the knowledge regarding incidence and risk factors of VTE in tibial plateau fractures is limited. This study aimed to investigate the incidence and risk factors of clinically important venous thromboembolism (CIVTE) in patients with tibial plateau fractures. All adult patients who underwent surgical treatment of tibia plateau fractures between 2003 and 2018 in our level 1 trauma center were included in the study. All patients suspected CIVTE were assessed by the ultrasonography and/or CT scan. Univariate and multivariate analysis were used to evaluate the association between potential risk factors and CIVTE Variables. Thirty-nine of 462 patients (8.4%) developed clinically important venous thromboembolism, in which pulmonary embolism (PE) and deep vein thrombosis (DVT) were observed in 18 (3.9%) and 21 (4.54%) patients, respectively. Male gender (OR 9.75; 95% CI 2.34–40.66), spine injury (OR 9.51; 95% CI 3.39–26.64), other extremity injury (OR 3.7; 95% CI 1.58–8.66), length of stay in ICU (OR 1.14; 95% CI 1.09–1.2) were all risk factors for CIVTE. The incidence of CIVTE in tibial plateau fracture was relatively high (8.4%); The male gender, spine injury, other extremity injury, length of stay in ICU were the independent risk factors.

## Introduction

Venous thromboembolism (VTE), which consists of deep venous thrombosis (DVT) and pulmonary embolism (PE) is a severe problem in operatively treated trauma and orthopedic patients^1,2^. Most of the literature on VTE is in hip and knee arthroplasty. The incidence of DVT ranges from 40 to 80% in major orthopedic procedures and the clinical PE from 4 to 10%^3,4^. There is sufficient evidence in the literature now to support the use of chemical prophylaxis for VTE in major orthopedic procedures. VTE in patients with fractures of tibia and distal bones is recently had more attention^5^. The importance of VTE prevention in foot and ankle surgery has also been emphasized^6–8^.

Usually, the fractures distal to the knee were recognized as a low risk of VTE; and there are no guidelines to support the use of chemical thromboprophylaxis for fractures around and distal to the knee. The clinical practice of VTE prophylaxis in tibial plateau fractures is subject to controversy^9^. Evidence-based thromboprophylaxis guidelines are not available for tibial plateau fractures. Even the American College of Chest Physicians does not provide recommendations for DVT prophylaxis after tibial plateau fractures^9^. In recent years, the practice has shifted from routine thromboprophylaxis of any isolated lower extremity DVT toward therapy for CIVTE^9,10^.

Interestingly, there are few studies on VTE associated with tibia plateau fractures. There is a large variation in the incidence of DVT (total incidence of DVT included asymptomatic and symptomatic DVT) in patients surgically treated for tibial plateau fractures and ranges from 1.7 to 42.9%^2,11^. It may be asymptomatic and mostly located in the distal to the knee^12^. The reported rates of PE range from 0 to 2.4%^11,13^. Moreover, most previous studies lack details regarding identifying risk factors associated with DVT and PE. Compared with the silent VTE, the CIVTE^5,14^ (defined as pulmonary embolism, proximal DVT, and/or symptomatic distal DVT) deserves more attention. However, to our best knowledge, there was no previous study on CIVTE in tibial plateau fractures. This study aimed to investigate the incidence and risk factors of CIVTE in surgically treated tibial plateau fractures.

## Patients and methods

The single-center retrospective cohort study was conducted at the University of California, San Francisco, USA. The Institutional Review Board approved this study and waived the requirement for informed consent because of the minimal risk to participants (IRB Number: 18-24291). All medical records and radiological studies of surgically treated tibial plateau fractures from Jun 2003 through April 2018 were reviewed. The inclusion criteria were as follows: (1) age older than 18 years, (2) patients with tibial plateau fractures, (3) the tibial plateau fracture treated operatively. Exclusion criteria were: (1) pathological fractures, (2) anticoagulation within 7 days before the injury, (3) the tibial plateau fracture treated non-operatively (4) prior VTE. The diagnosis of tibial plateau fracture was identified according to the diagnosis codes in the hospital discharge registry. The surgical treatment was checked from surgical record.

Chemical thromboprophylaxis using subcutaneous low molecular weight heparin (LMWH) (enoxaparin at prophylactic dose) for 14 days was routinely applied. LMWH was stopped 12 h before surgery and restarted 12 h after surgery. After 14 days, the thromboprophylaxis was p.o. rivaroxaban or warfarin for 4 weeks or more. For the patients with contraindications to chemical anticoagulation, an inferior vena cava filter (IVCF) was placed.

During hospitalization, the patients underwent routine VTE surveillance, which means signs and symptoms of VTE were checked by the doctor during their hospital stay daily. But patients did not undergo routine duplex ultrasound (DUS, for extremity DVT) or spiral CT (for PE); they were only checked by DUS or spiral CT when there was clinical suspicion (The patients were suspected that VTE or PE was developed when they suffered an acute, severe and sudden pain, severe swelling in an injured limb or sudden and severe cough, dyspnea.) for VTE. Proximal DVT or PE can be confirmed at any timepoint from injury to hospital discharge via radiographic methods and was defined as CIVTE. In our study, the follow-up stopped at hospital discharge. All DUS were performed by a certified ultrasonographer and interpreted by an attending radiologist. Similarly, DUS of upper extremity was performed when clinical suspicion of DVT was raised. When CIVTE was diagnosed, treatment using LMWH (enoxaparin at treatment dose) was started and continued for 14 days. In patients with the contraindications to anticoagulation, and if CIVTE was diagnosed, a retrievable IVCF was placed.

The following data were collected: Age, gender, medical comorbidities, history of VTE, mechanism of injury, fracture classification (open vs. closed, Schatzker classification), time from injury to the first surgery, time from injury to definitive surgery (if temporary ex-fix applied), associated injuries (head, chest, abdomen, other orthopedic injuries), length of stay in ICU (if any), VTE prophylaxis, Duplex Ultrasound and/or spiral chest CT scan for assessment of VTE (if performed), location of VTE, the length of hospital stay. Clinical data were recorded by two investigators from medical records.

### Statistical analysis

Continuous variables data were presented as means and standard deviations. Student t-test was utilized for continuous variables. For comparisons between VTE group and no VTE group, the Chi-square and Fisher exact tests were utilized for the categorical variables. After univariate analysis, multivariate logistic regression analysis was performed to identify the independent risk factors between various risk factors. A p value of < 0.05 was defined as statistical significance. The Statistical Package for Social Sciences (SPSS) software version 19.0 (IBM, Chicago, IL, USA) was used.

### Ethical approval

All procedures performed in studies involving human participants were in accordance with the ethical standards of the institutional and/or national research committee and with the 1964 Helsinki declaration and its later amendments or comparable ethical standards. This study was approved by the Institutional Review Committee of University of California, San Francisco (IRB Number: 18-24291). The informed consent was waived because of the minimal risk to participants.

### Informed consent

Given the retrospective nature of the study it was not deemed necessary to seek informed consent from the patients whose data was used. There is no patient identifiable data in this manuscript.

## Results

### Patient characteristics

Over the study period, 513 patients were included according to inclusion criteria while 51 patients were excluded according to exclusion criteria, of which 27 patients met prior VTE, 13 patients met anticoagulation within 7 days before the injury, 7 patients met the tibial plateau fracture treated non-operatively, and 4 patients met pathological fractures. Finally, 462 patients that met inclusion criteria were included. The mean age was 46.2 ± 15.4 years (range: 18–88), 309 were males, and 153 were females. The demographic data are shown in Table 1. According to the Schatzker classification, 249 patients sustained low energy injury (Type 1–3), and 213 patients sustained high energy injury (Type 4–6). 161 patients underwent staged surgeries. The average time from injury to definite fixation was 8.4 ± 8.1 days, and the average time from surgery to discharge was 9.6 ± 14.1 days. (Table 1) VTE prophylaxis was administered for 349 patients (75.5%). It was started at hospital admission and continued until 14 days post-operatively. 311 patients (67.3%) received LMWH, 7 patients (1.52%) received heparin, 7 patients (1.52%) received aspirin, 11 patients transitioned from enoxaparin to warfarin, 13 patients (2.81%) received IVCF. 113 patients (24.5%) did not receive any prophylaxis. (Table 2).

**Table 1:** Summary of demographic data with comparison of group with and without CIVTE.

Variables	No CIVTE	CIVTE	Total
**Gender**			
Male	274 (64.8%)	35 (89.7%)	309 (66.9%)
Female	149 (35.2%)	4 (10.3%)	153 (33.1%)
Age	46.7 ± 15.0	50.8 ± 16.4	46.2 ± 15.4
BMI	27.2 ± 5.1	26.2 ± 5.0	27.2 ± 5.2
**Open fracture**			
Yes	34 (8.0%)	8 (20.5%)	42
No	389 (92%)	31 (79.5%)	420
**Mechanism**			
PVA	153 (36.2%)	16 (41%)	169 (36.6%)
MVA	87 (20.6%)	10 (25.6%)	97 (21%)
Fall	130 (30.7%)	10 (25.6%)	140 (30%)
GSW	5 (1.2%)	1 (2.6%)	6 (1.3%)
Other	48 (11.3%)	2 (5.1%)	50 (10.8%)
**Classification**			
I–III	237 (56.0%)	12 (30.8%)	249
IV–VI	186 (42.3%)	27 (69.2%)	213
**Associated injuries**			
Traumatic brain injury	13 (3.1%)	11 (28.2%)	24
Chest injury	23 (5.4%)	9 (23.1%)	32
Abdomen injury	13 (3.1%)	4 (10.3%)	17
Pelvic fractures	27 (6.4%)	10 (25.6%)	37
Spine injury	13 (3.1%)	15 (38.9%)	28 (6%)
Extremity injury	104 (24.6%)	23 (59.0%)	127 (27.5%)
Compartment syndrome	32 (7.6%)	4 (10.3%)	36
**Comorbidities**			
HTN	92 (21.7%)	8 (20.5%)	100
DM	30 (7.1%)	2 (5.1%)	32
Heart disease	11 (2.6%)	3 (7.7%)	14
HLD	32 (7.6%)	3 (7.7%)	35
COPD	5 (1.2%)	3 (7.7%)	8
Asthma	14 (3.3%)	1 (2.6%)	15
Previous VTE	6 (1.4%)	2 (5.1%)	8
Cancer	8 (1.9%)	5 (12.8%)	13
**Smoking**			
Yes	127 (30.0%)	13 (33.3%)	140
No	71 (16.8%)	1 (2.6%)	72
Unknown	225 (53.2%)	25 (64.1%)	250
**ETOH**			
Yes	165 (12.3%)	10 (25.7%)	175
No	52 (39.0%)	3 (7.7%)	55
Unknown	206 (48.7%)	26 (66.7%)	232
**Illicit drug use**			
Yes	106 (25.1%)	9 (23.1%)	115
No	72 (17.0%)	2 (5.1%)	74
Unknown	245 (57.9%)	28 (71.8%)	273
**Fixation method**			
One-staged	287 (67.8%)	14 (35.9%)	301
Two-staged	136 (32.2%)	25 (64.1%)	161
**Tourniquet**			
Yes	239 (56.5%)	15 (38.5%)	254
No	184 (43.5%)	24 (61. 5%)	208
**ICU**			
Yes	34 (8.0%)	24 (38.5%)	58
No	389 (92%)	15 (61.5%)	404
Injury to definitive surgery	7.9 ± 7.6	13.7 ± 10.6	8.4 ± 8.1
Surgery to discharge	8.5 ± 12.5	21.7 ± 22.5	9.6 ± 14.1
Length stay in ICU	0.9 ± 4.0	10.3 ± 14.2	14.3 ± 11.5
hospital stay (Median; Min–Max)	13 (1–134)	28.5 (4–118)	17.9 ± 16.3
**Prophylaxis**			
Yes	313 (74%)	36 (92.3%)	349 (75.5%)
No	110 (26.0%)	3 (7.7%)	113 (24.5%)

*PVA* pedestrian VS motor vehicle accident, *MVA* motor vs motor accident, *GSW* gunshot wound, *HTN* hypertension, *DM* diabetes mellitus, *HLD* hyperlipidemia, *COPD*: chronic obstructive pulmonary disease, *ETOH* ethyl alcohol, *ICU* Intensive Care Unit.

**Table 2:** VTE prophylaxis and therapeutic methods.

Medication with/without IVCF	Prophylaxis	Treatment
Enoxaparin	30 mg–60 mg daily	> 80 mg daily
Heparin	NA	5000U bid–5000 tid
Warfarin	NA	Adjusted based on INR
Aspirin	81–325 mg daily	NA
Enoxaparin/IVCF	30 mg–60 mg daily/IVCF	80–120 mg daily/IVF

### Incidence of CIVTE

Of the 462 patients, 39 patients (8.4%) developed CIVTE, including 21 (4.5%) patients with proximal DVT and 18 patients (3.9%) with PE. Of the 21 patients with DVT, proximal lower extremity DVT was detected in 13 patients (2.9%), and proximal upper extremity DVT was detected in 8 patients (1.7%). The 18 patients with PE (including 2 patients developed PE combined with DVT) underwent placement of the IVCF, followed by anticoagulant therapy. The IVCFs were retrieved before discharge (Table 3). Two patients died due to multiple system organ failures. No fatal PE occurred, and no major bleeding events or bleeding into a critical site was diagnosed.

**Table 3:** Incidence of CIVTE.

Variable	N (%)
VTE	39 (8.44%)
DVT	21 (4.55%)
Upper extremity DVT	8 (1.73%)
Lower extremity DVT*	13 (2.81%)
PE	18 (3.90%)
DVT with PE**	2 (0.43%)

*All the lower extremity DVT located in the proximal veins.

**DVT with PE was included in PE.

### Risk factors for DVT

When patients who developed CIVTE were compared to patients who did not develop CIVTE, there was no significant difference regarding age, BMI, smoking, ETOH dependence, illicit drug use, medical comorbidity (HTN, DM, Heart disease, HLD, Asthma, Previous VTE), mechanism, abdominal injury or not, compartment syndrome or not, length of hospital stay, between patients with DVT and without DVT (p > 0.05). After univariate analysis, the factors of p > 0.05 were removed, while the important clinical factor (age) of p > 0.05 were included in multivariate logistic regression analysis. Multivariate logistic regression analysis showed that male gender, patients with associated injuries (spine injury, extremity fractures), and the length of stay in ICU were independent risk factors for VTE in tibial plateau fractures (Table 4).

**Table 4:** Multivariate logistic regression analysis of risk factors of CIVTE.

	Exp (β)/OR	95%CI	
Gender (male:female)	9.75	2.34	40.66
Associated spine injury	9.51	3.39	26.64
Associated extremity injury	3.7	1.58	8.66
Length stay in ICU	1.14	1.09	1.2

*Exp (β)* exponentiation of the β coefficient, *CI* Confidence Interval. The OR for Length stay in ICU refers to precent of VTE increasing per 1 day.

## Discussion

The current study showed that the incidence of CIVTE in tibial plateau fracture was 8.4%, which was higher than previous reports^2,11–13,15–18^(Table 5). We found that the factors associated with CIVTE were male gender, the presence of spine injury, the presence of other extremity injury, length of stay in ICU.To the best of our knowledge, this is the most extensive study to investigate the incidence and risk factors of CIVTE focused on patients with tibial plateau fractures.

**Table 5:** Comparison of the current study with previous studies on VTE in tibia plateau fractures.

Author	Current study	Stamer DT	Abelseth, G	Ebraheim NA	David P. Barei	Stephen Andrew Sems	Nikolaos Manidakis	Lapidus	Hu Wang
Year	2018	1994	1996	2004	2006	2009	2010	2013	2018
No	462	22	102	117	41	136/151	125	45,968	1825
Tibial plateau	462	23	28	117	41	62	125	395	176
VTE	39							15 (3.8%; 95%CI 2.3–6.3)	
DVT	18	1	12	2	8	3^#^	7^#^	13 (3.3%; 95% CI 1.9–5.7)	41 (preoperative) 46 (postoperative)
PE	21				0		3 in 7^#^	3 (0.8%; 95% CI 0.2–2.4)	1
FPE	0							2 (0.5%; 95% CI 0.1–2.0)	
Incident of VTE*	8.4%	4.3%*	42.9%	1.7%*	19.5%*	4.8%*	5.6%*	3.8%*	26.7%
Location of DVT									
proximal	13(5in upper limb		0		5	2		Cannot identify	6 (3.4%) 8 (4.5%) preoperative
Distal			12		3	1			36 (20.5%) (preoperative) 72 (40.9%) (posteoperative)
Prophylaxis	LMWH, warfarin,Aspirin, IVCF			NA	warfarin or LMWH	LMWH	NA	LMWH	LMWH

The comparisons were informality.

^#^The number of DVT or PE were not the accurate.

*The incidences of VTE were not provided by authors. These incidences were calculated by us according the data from original literature.

Previous studies reported that the DVT (asymptomatic and symptomatic) rate was as high as 77% in patients with lower extremity fractures, of which asymptomatic DVT mainly contributes to a high proportion^1,2,19^. The risk factors for DVT are well reported in joint replacement, polytrauma, and certain fractures. Surprisingly, limited information is available about VTE in patients with tibial plateau fractures, and the reported rates of VTE are highly variable (Table 5). Most of the studies mentioned VTE only as a minor complication of the tibial plateau fractures. None of them specifically analyzed the VTE in tibial plateau fractures, which may reduce the power of the evidence. In a recent study, Wang et al. found the incidence of proximal DVT in tibial plateau fractures to be 4.5% after a routine screening of bilateral lower extremities. As all DVT (symptomatic and asymptomatic) were included, it is probably an overestimation of CIVTE^12^.

### The incidence of CIVTE

75.5% (349/462) of the patients were received chemoprophylaxis. A total of 23 positive DUS and 18 positive CT were observed in 39 patients, putting the overall incidence of CIVTE at 8.4%. The incidence of CIVTE in chemoprophylaxis group was 10.3%, (36/349). To our best knowadge, this study was the first one reported the cumulative incidence rate of CIVTE rather than pervious reported the total incidence of VTE (slient DVT + symptomatic VTE)^2^. But in our study the overall of incidence of CIVTE may underestimation as the short-term surveillance missing the potential positive patients who would develop CIVTE after discharge from hospital. On the other hand, our study only included 462 patients, which is small to analyze the incidence of VTE. Therefore, the real results may be higher than our observation.

### The risk factors of DVT in tibial plateau fractures

We identified four independent risk factors for VTE in surgically treated tibial plateau fractures: male gender, spine injury, other extremity injury, length of stay in ICU.

The current study found that gender was an independent risk factor for VTE. This may be due to more males being injured and enrolled in the current study. In the study by Silverstein et al., the incidence of VTE was higher in women during the childbearing years, it was higher in men older than 45 years old. The overall age-adjusted incidence rate was 130 per 100,000 per year (95% CI 121–138) in males and 110 per 100,000 (95% CI 104–116) in females (male–female ratio, 1.2:1)^20^. In the current study, the overall mean age of the patients was 46.2 ± 15.4 years. The rate of CIVTE in males and females was 11.3% and 2.6%, respectively. Further analysis showed that male patients with tibial plateau fractures had an 11.3-fold increased risk of CIVTE.

The length of stay in ICU as a high risk of VTE might be due to multiple factors such as mechanical ventilation, vasopressor use, and central venous catheter use^21^. The length stay in ICU was an independent risk factor of CIVTE in the current study. CIVTE was detected 41.4% (24/58) of the ICU patients and average length of stay was 14.3 ± 11.5 days. The incidence of CIVTE was much higher than previously reported, which could partially be explained by longer length of stay in ICU^22^. Malinoski et al.^23^ performed a retrospective study with 411 patients with a mean length of stay in ICU of 8 ± 9 days. They reported that ICU patients with extremity injury and without chemical thromboprophylaxis were an independent predictor of VTE, with 2.4-fold increase in the risk. On the other hand, a longer of stay in the ICU may mean high energy injury or severe soft tissue injury and a longer time of immobilization and bed rest until definitive fixation. Park et al. reported that compared with the low energy, the high energy hip fractures showed a 2.451-fold increased risk of VTE (OR 2.5; 95% CI 1.2–4.9)^24^.

In previous studies, orthopedic, neurological, and pelvic surgeries has been associated with an especially high incidence of VTE^25^. Similarly, the current study showed associated spine injury, and associated extremity injury were the independent risk factors of CIVTE in tibial plateau fractures. The analysis revealed that the associated with spine injury and was the more significant risk factors of CIVTE, (Odds ratio 9.5). These might be due to the delay in chemical thromboprophylaxis. Although recent studies^26–28^ have advocated that the safety of earlier and more aggressive prophylaxis to reduce the risk of VTE, physicians are hesitant to initiate chemical prophylaxis due to the potential risk of hemorrhage in these patients. Stawicki et al^29^ showed that the incidence of DVT in multiple injured patients was higher than the isolated lower extremity fractures. Similarly, Park et al. reported that prolonged hospitalization after multiple fractures might be a risk factor of the development of VTE^30^. Consistent with these prior studies, in the current study, the rate of VTE was higher in patients with associated extremity fractures than those with isolated fractures.

### Limitations

Our study has several limitations due to its retrospective nature such as selection bias. Some predictive variables may not have sufficient statistical power due to the smaller number of patients in subgroups. Additionally, the period of the study was relatively long, and the guidelines and practice of VTE prophylaxis had variations. Our study may overestimate the correlation between length of hospitalization and morbidity of VTE as the patient stay longer in hospital and have more chance of detecting VTE, while in our study, we can not detect and record if the patient who was discharged earlier, has VTE or not after discharged.

## Conclusion

The incidence of CIVTE in tibial plateau fracture was high (8.4%); The male gender, associated spine injury or extremity injury, length stayed in ICU were the independent risk factors.

## Data Availability

Data is available on reasonable request via contacting the corresponding authors.
